# Case Report: Benign myoepithelioma presenting as fever of unknown origin: a rare clinical manifestation of an uncommon tumor

**DOI:** 10.3389/fonc.2026.1784983

**Published:** 2026-03-13

**Authors:** Yiran Shou, Congwei Jia, Xiaotong Tian, Di Wu, Ling Luo, Taisheng Li, Li Zhang, Zhengyin Liu

**Affiliations:** 1 Department of Infectious Diseases, Peking Union Medical College Hospital, Beijing, China; 2 Department of Pathology, Peking Union Medical College Hospital, Beijing, China; 3 Department of Intensive Care Unit, Beijing Hospital, National Center of Gerontology, Institute of Geriatric Medicine, Chinese Academy of Medical Sciences, Beijing, China; 4 Department of Rheumatology, Peking Union Medical College Hospital, Beijing, China

**Keywords:** fever of unknown origin, foot mass, myoepithelioma, soft tissue tumor, surgery

## Abstract

The etiology of fever of unknown origin (FUO) is complex and diverse, with benign neoplasms remaining a rarely reported cause. This case report describes a young female patient who presented with FUO and persistently elevated inflammatory markers. A comprehensive diagnostic workup revealed no clear evidence of infectious, autoimmune, or malignant disease. However, during hospitalization, a previously stable, painless mass on her left foot, which presented for two years, exhibited increased local skin temperature. Imaging studies identified a metabolically active subcutaneous lesion at the site. The patient’s condition improved rapidly following excision of the mass, and histopathological examination confirmed the diagnosis of soft tissue myoepithelioma. Soft tissue myoepithelioma, a rare neoplasm of uncertain differentiation, was identified as the underlying etiology of the patient’s clinical presentation. Typically characterized by an indolent and subtle clinical course, this neoplasm most commonly manifests as a painless mass in the extremities or trunk. The disease exhibits a roughly equal sex distribution, with a peak incidence occurring between the ages of 30 and 40 years. Histologically, soft tissue myoepithelioma demonstrates a heterogeneous composition of myoepithelial cell types, predominantly epithelioid cells, and exhibits immunohistochemical expression of both epithelial and myogenic lineage markers. It is noteworthy that fever associated with myoepithelioma is extremely rare, with very few cases reported in the literature to date. This case highlights the rare presentation of soft tissue myoepithelioma as a hyperinflammatory fever, thereby expanding the recognized clinical spectrum of this tumor and deepening the understanding of this distinct entity.

## 1 Introduction

While malignancy is a known cause of fever of unknown origin (FUO), persistent high fever and a hyperinflammatory state caused by benign neoplasms are rarely documented (1). Myoepithelioma is a benign tumor with an indolent growth pattern and is seldom reported to cause febrile presentations.

This report describes a rare case of FUO caused by a pedal myoepithelioma. The patient achieved complete clinical remission following complete surgical excision of the tumor and remained stable with sustained remission at the one-year follow-up.

## 2 Case description

### 2.1 Admission status

A 23-yeaer-old female developed daily fever following a forest excursion, typically in the afternoon, without associated symptoms. Her febrile symptoms showed no response to a two-week empirical course of moxifloxacin, even demonstrating a progressive increase in peak temperature, reaching 39.2 °C. Lab results showed normal white cell and neutrophil counts with mild anemia, normal renal and liver function, and markedly elevated inflammatory markers including C-reactive protein (CRP), erythrocyte sedimentation rate (ESR) and ferritin. Serological tests showed no clear evidence of common bacterial, viral for fungal infections. Though antinuclear antibody (ANA) was positive at low titer, other autoimmune panels were negative. Physical examination showed that multiple small lymph nodes were palpable in bilateral cervical and bilateral inguinal regions, with the largest node located in the left inguinal area. A soft, linear, reddish, cystic mass was noted on the left buttock. A non-tender cystic mass, approximately 2 cm in diameter, was palpable on the medial aspect of the left ankle.

#### 2.2 Relevant history and past medical history

The fever began on the same day after visiting a wooded suburban area, while the patient denied any history of tick bites or recent animal contact. She had a long-standing painless subcutaneous nodule on the left buttock over 10 years, previously assessed by dermatology as a dermoid cyst. She also had a history of painless cystic swellings on the left wrist and medial left ankle for 2 years. While the wrist swelling resolved with massage therapy, the ankle swelling has remained stable in size during the course of fever.

### 2.3 Laboratory and imaging findings

Complete Blood Count (CBC): White blood cell count and differential count were within normal limits. Hemoglobin gradually decreased to 89 g/L. Platelet count was significantly elevated.

Renal and Liver Function Tests, Urinalysis, and Stool Routine: All within normal ranges.

Markedly Elevated Inflammatory Markers: C-reactive protein (CRP) 157.87mg/L, sedimentation rate (ESR) 104mm/h, SF 537ng/L. PCT was remained within normal limits.

Infectious Disease Workup: Three sets of blood cultures and urine culture were negative (1–3). -β-D-glucan assay, T-SPOT.TB, and Widal/Weil-Felix tests showed no positive findings.

Autoimmune Evaluation: Antinuclear antibody (ANA) was positive at a titer of 1:80. Antiphospholipid antibodies, rheumatoid arthritis antibody panel, and systemic vasculitis antibody panel were all negative. Coombs test and lactate dehydrogenase (LDH) levels were within normal limits. The levels of IL-6 and IL-8 were significantly elevated, measuring 53.4 pg/ml and 126 pg/ml respectively, while IL-10 was within the normal range. The TNF-α level was mildly increased at 9.3 pg/ml.

Oncologic Screening: Tumor markers were negative. No M-protein was detected in blood or urine. Bone marrow examination revealed no evidence of hematologic malignancy.

Imaging Studies: Ultrasound and MRI indicated multiple enlarged lymph nodes in bilateral inguinal regions, along the iliac vessels, and within the pelvis. Echocardiography showed no evidence of valvular vegetations. Chest and abdominal CT scans, as well as renal and gynecological ultrasounds, revealed no significant abnormalities. PET/CT scan revealed multiple metabolically active lymph nodes (increased uptake) in the left iliac region, left inguinal area, mesentery, and retroperitoneum, with the most pronounced uptake (SUVmax5.2) in the left inguinal and left external iliac vascular regions.

### 2.4 Lymph node biopsy and initial surgery

Given that multiple imaging findings indicated abnormally enlarged lymph nodes, a biopsy was performed on the largest and most metabolically active left inguinal lymph node. All pathogen-specific tests on the lymph node tissue were negative, including metagenomic next-generation sequencing (mNGS). Histopathological examination of the lymph node revealed reactive hyperplasia. In summary, the lymph node biopsy did not provide definitive evidence of an infectious disease or malignancy.

Although the buttock mass had a long history and showed no significant changes during the recent febrile illness, it was considered necessary to thoroughly rule out a potential association, considering the abnormal lymph nodes were located within its lymphatic drainage area. Therefore, the left buttock mass was surgically excised by plastic surgeon. Pathogen-specific tests on the excised mass were negative. Histopathological examination confirmed the mass to be an epidermoid cyst.

### 2.5 Diagnostic turn in the course

At this stage, a multidisciplinary discussion was convened to collectively review the existing evidence. Given the patient’s history of exposure to a forested area, the possibility of a vector-borne infection was considered. A one-week empirical trial of minocycline was administered to cover atypical pathogens, but no clinical response was observed. Based on the comprehensive negative workup for infectious etiologies and the lack of response to broad-spectrum antibiotic therapy, the likelihood of an active infectious disease was considered low. Furthermore, a thorough evaluation of imaging studies, bone marrow examination, and histopathological biopsy findings revealed no evidence of solid organ malignancy or hematologic neoplasms such as lymphoma. Considering the clinical presentation of prolonged fever, diffuse skeletal involvement on imaging, and a hyper-inflammatory state, the rheumatology consultant raised the possibility of Adult-Onset Still’s Disease (AOSD). However, the absence of the classic rash and the lack of improvement in both fever and inflammatory markers after a one-month trial of corticosteroid therapy cast significant doubt on this diagnosis. The etiology of the patient’s condition still remained under investigation.

**Figure 1:**
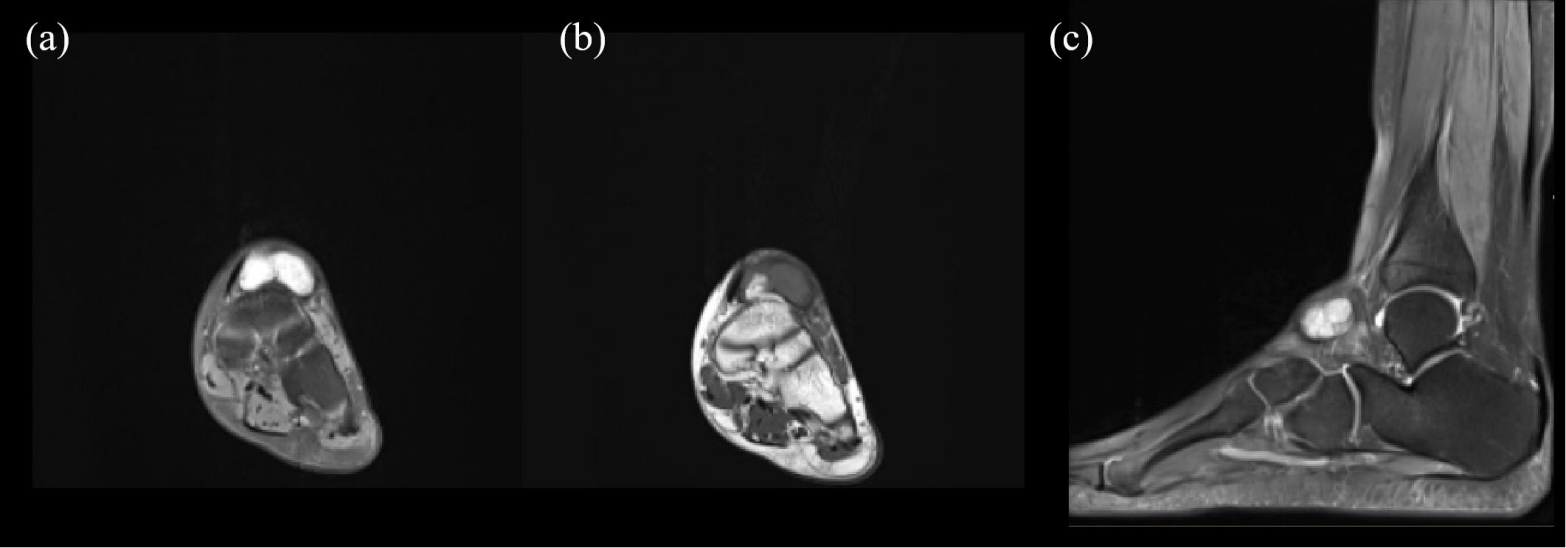
MRI of the ankle reveals a subcutaneous lesion in the left ankle. **(a, b)** A long T1 and long T2 signal is observed in the subcutaneous tissue of the dorsal foot. **(c)** Fat-suppressed sequence showed well-defined boundaries with internal septations, displayed from a lateral perspective.

**Figure 2:**
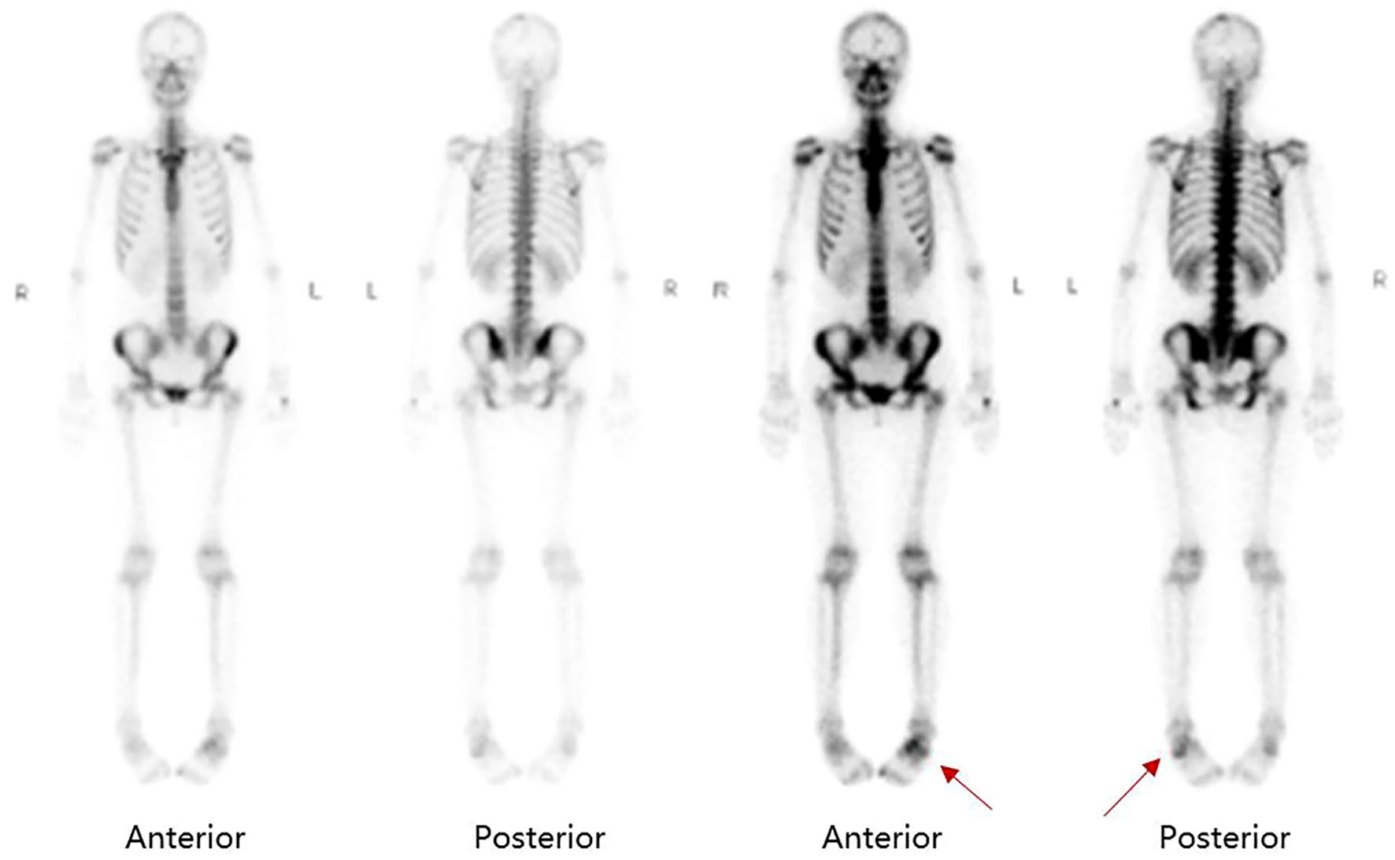
Whole-body bone scan showed increased radiotracer uptake in the left tarsal bones and the left first toe (as indicated with red arrows).

By this point, the patient had been symptomatic for over five months without a definitive diagnosis, and both her fever and inflammatory markers remained persistently elevated. A turning point occurred during a routine daily physical examination when the attending physician noted increased local skin temperature over the mass on her left ankle, although there was no significant redness or pain. Upon further questioning, the patient recalled a brief episode of redness and swelling in the same area about a year prior, which had resolved spontaneously. Further evaluation of the left ankle mass was pursued. MRI revealed a subcutaneous cystic lesion on the dorsum of the left foot, exhibiting long T1 and long T2 signals with internal septations (**Figure 1**). Consequently, a whole-body bone scan was performed to further evaluate the localized lesion, which observed diffusely increased radiotracer uptake in the axial skeleton, along with areas of abnormally increased radiotracer concentration in the left tarsal bones and the left first toe (**Figure 2**).

**Figure 3:**
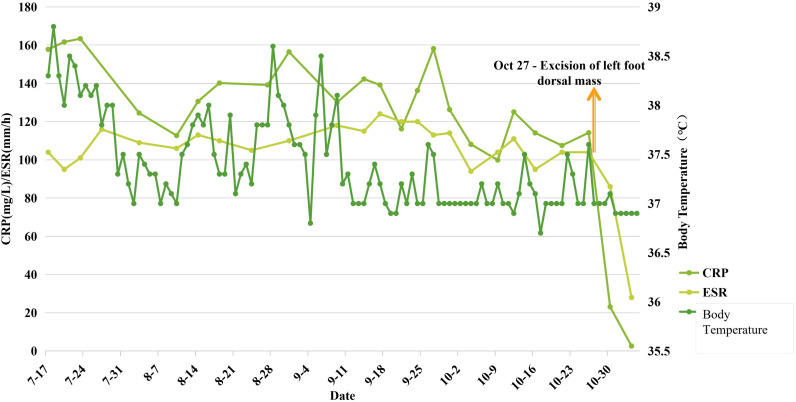
Line graph of changes in body temperature and inflammatory markers before and after excision of the left foot mass. The graph shows that preoperatively even with the regular administration of NSAIDs, body temperature continued to fluctuate and remained elevated above the normal baseline, while CRP and ESR levels stayed persistently high. Postoperatively, body temperature normalized and stabilized, and the inflammatory markers declined rapidly.

**Figure 4:**
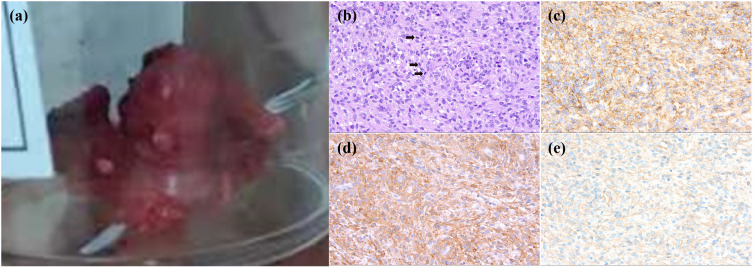
Gross and microscopic features of the left foot mass. **(a)** Appearance of the excised mass showing irregular solid soft tissue with medium texture. **(b)** HE staining (×400) showed that the tumor cells exhibited a mixed morphology of oval and short spindle-shaped cells, arranged in bundles or an interwoven pattern, with active growth and visible mitotic figures (as indicated by red hollow arrows).as indicated by the red hollow arrows. **(c)** Epithelial marker AE1/AE3 staining (×400). **(d)** Myoepithelial marker SMA staining (×400). **(e)** Myoepithelial marker calponin staining (×400).

Orthopedic consultants recommended excision of the mass for definitive pathological diagnosis. The mass was subsequently excised under local anesthesia. Remarkably, within three days post-operatively, the patient’s fever subsided and her inflammatory markers normalized rapidly (**Figure 3**). The excised left foot mass measured 4.0 x 2.2 x 1.8 cm on gross examination, demonstrating an intermixed gray-pink and gray-yellow cut surface with firm consistency (**Figure 4**). Microscopic examination showed a tumor composed of short spindle-shaped cells arranged in fascicles or an interlacing pattern, with demonstration of moderate proliferative activity and identifiable mitotic figures. Immunohistochemical staining demonstrated positivity for the epithelial marker AE1/AE3 as well as the myoepithelial markers calponin and SMA. These immunohistochemical results demonstrate expression of epithelial markers, which have a high positive rate in myoepithelial tumors (2). Ki67 is positive in 15% of tumor cells. Tumor cells were negative for CD99, GPAF, S100, CD 34, CD 31, as well as desmin and Myo-D1. At the one-year follow-up, the patient demonstrated clinical stability, with neither recurrence of fever nor evidence of tumor recurrence on follow-up foot MRI.

## 3 Discussion

Myoepithelioma is a rare neoplasm, typically considered to originate from salivary glands. According to the WHO classification, myoepithelioma is categorized alongside myoepithelial carcinoma and mixed tumors under the group of tumors of uncertain differentiation (3). Soft tissue myoepithelioma represents an even rarer subtype within this category.

Soft tissue myoepithelioma most commonly occurs in the extremities or trunk, followed by head and neck (2). The lesions are often located in the superficial subcutaneous layer, although cases involving deeper soft tissues such as intramuscular, intermuscular, or subfascial locations, have also been reported (4). The clinical presentation of soft tissue myoepithelioma in the extremities can resemble that of dermatofibroma or melanoma (5), posing a diagnostic challenge.

Regarding clinical presentation, a painless mass is reported to be the most common symptom of soft tissue myoepithelioma. Some patients may present with swelling or pain due to mass effect (3, 6). Fever and a hyperinflammatory state associated with myoepithelioma are exceedingly rare. A literature review identified only one reported case of pulmonary myoepithelioma, which similarly presented with fever and markedly elevated inflammatory markers in the absence of significant respiratory symptoms (7). This represents the only published case report we have identified with clinical presentations comparable to our case. Neoplastic fever predominantly occurs in hematologic malignancies and advanced solid tumors (8). Its pathophysiology is attributed to the release of pyrogens by tumor cells, such as TNF, IL-1, IL-6, and IFN and/or the activation of the immune system leading to inflammatory mediator release. The naproxen test is a classical diagnostic tool for neoplastic fever, characterized by fever unresponsive to empirical broad-spectrum antibiotics but showing rapid and sustained defervescence with NSAIDs, a pattern consistent with the therapeutic response observed in our patient (9). Given the rarity of reported fever associated with myoepithelioma, the underlying pathophysiology remains speculative. IL-6 is well-documented to play a central role in both the initiation and maintenance of fever, as well as in driving the acute-phase response by inducing the production of various acute-phase proteins (10). Furthermore, IL-6 has been reported to act as an autocrine growth factor during the differentiation of pleomorphic adenoma cell lines toward a myoepithelial phenotype (11), suggesting the potential for IL-6 production by such tumors. Thus, we hypothesize that during the growth of the foot myoepithelioma in this patient, tumor cells spontaneously produced IL-6, which entered the systemic circulation. Once IL-6 accumulated to a critical threshold, it likely triggered recurrent fever and a hyperinflammatory state. However, this hypothesis hinges on IL-6, and potentially other key pyrogens such as TNF and IL-1, being produced by the tumor itself. Clues were identified upon review of the patient’s cytokine levels, which revealed a markedly elevated IL-6 level after admission. However, it is regrettable that cytokine levels were not rechecked postoperatively, precluding a comparison with the preoperative values. This constitutes a limitation of this report. To validate this theory, further work is required, including confirming the expression of IL-6 and other key pyrogenic cytokines within the tumor tissue, conducting *in vitro* functional assays to establish causal relationships, and performing clinical correlative studies by longitudinally monitoring serum cytokine levels, inflammatory markers, and body temperature in a larger patient cohort.

Imaging features of benign myoepithelioma on ^18^F-FDG PET/CT typically show no significant metabolic uptake (7, 12), whereas malignant myoepithelial tumors at various sites (including lung, bone, pelvis, and parapharyngeal space) are characterized by significantly increased metabolic activity, reflected in high SUVmax values (13–16). There are no established reports describing the appearance of myoepithelioma on whole-body bone scan. Furthermore, the utility of this imaging modality in differentiating benign from malignant forms remains undocumented.

Tumor size is highly variable. Histologically, myoepithelioma exhibits complex composition, often containing at least one type of myoepithelial-derived cell—such as epithelioid, clear, spindle, or plasmacytoid cells. The diagnosis of malignancy is primarily predicated on pathological examination. Pathological examination remains the main standard for determining malignancy. Based on the degree of cellular atypia, these tumors are classified as benign (myoepithelioma or mixed tumor), showing low grade or no atypia, or malignant (myoepithelial carcinoma or malignant mixed tumor), exhibiting moderate to severe atypia, sometimes with necrosis (2). In salivary gland myoepithelioma, mitotic activity and invasive growth into surrounding tissues aid in distinguishing benign from malignant forms. However, no established cutoff for mitotic count has been defined.

The immunohistochemical markers also provide supportive diagnostic evidence. Epithelial markers (AE1/AE3, PAN-K, CAM 5.2) are typically positive. Myogenic markers calponin and SMA show high sensitivity and positivity, as demonstrated in this case. A dual nuclear and cytoplasmic positivity for S-100, combined with positivity for epithelial and myogenic markers, strongly supports a diagnosis of myoepithelioma. The nuclear marker p63 is positive in approximately one-fourth of cases; however, its expression is not specific as it may also occur in other tumor types (2). Therefore, its diagnostic utility relies on appropriate morphological context and co-expression of other markers, though p63 positivity can help exclude morphologically similar entities such as leiomyoma or schwannoma. Nuclear markers GFAP and SOX10 also show high positivity rates. Nevertheless, isolated case reports have indicated that some myoepitheliomas may exhibit negative expression for the aforementioned markers (17, 18), a pattern consistent with the immunohistochemical profile observed in the present case.

The primary treatment is wide excision, although cases treated with surgery combined with adjuvant chemotherapy, as well as chemotherapy alone for unresectable lesions, have been reported (6). Reported recurrence rates vary widely (17–50%), often associated with incomplete resection or positive surgical margins (2, 6, 19). Due to the rarity of this disease and the limited availability of large-scale cohort data, reliable long-term follow-up and prognostic information remain insufficient. Recurrence and even metastasis are primarily linked to positive resection margins, highlighting the limited understanding of the tumor’s malignant potential and emphasizing the importance of careful pathological evaluation, timely follow-up, and consideration of re-excision or sequential chemotherapy when indicated.

## 4 Conclusion

We present this case to highlight an exceptionally rare presentation of a soft tissue myoepithelioma manifesting as fever and a hyperinflammatory state. To our knowledge, this is the first reported case of a soft tissue myoepithelioma presenting as FUO, which makes this case particularly noteworthy.

This report aims to broaden the diagnostic perspective in the etiological diagnosis of FUO. Beyond the common etiologies such as malignancy, infectious diseases, or non-infectious autoimmune diseases, rare possibilities like this benign neoplasm should also be considered. The diagnostic process in this case demonstrated the critical importance of repeated physical examinations and sustained vigilance for new clinical signs throughout the course of illness. Clinicians must remain alert to evolving symptoms and recognize disease as a dynamic process. Moreover, a meticulous history proved crucial. As in this case, the patient’s recall of a subtle foot symptom one year prior ultimately guided the investigation toward the correct diagnosis.

Furthermore, this case expands the clinical spectrum of benign myoepithelial tumors. It suggests that such lesions may present with features not previously well recognized, reminding us that even histologically benign tumors can occasionally drive significant systemic inflammation. While we propose that tumor-secreted IL-6 may contribute to the observed fever and hyperinflammation, this study is limited by the absence of serial cytokine monitoring and experimental validation at the molecular or cellular level. This singular case underscores the importance of recognizing such atypical presentations and calls for the accumulation of similar cases to enable meaningful series analysis. Ultimately, a deeper investigation into the cytokine expression profiles of myoepitheliomas and their role in paraneoplastic inflammation is warranted to move from association to mechanism.

## Data Availability

The original contributions presented in the study are included in the article/supplementary material. Further inquiries can be directed to the corresponding authors.
